# Enhancement of ELM by Clustering Discrimination Manifold Regularization and Multiobjective FOA for Semisupervised Classification

**DOI:** 10.1155/2015/731494

**Published:** 2015-05-27

**Authors:** Qing Ye, Hao Pan, Changhua Liu

**Affiliations:** ^1^School of Computer Science and Technology, Wuhan University of Technology, Wuhan 430000, China; ^2^Yangtze University College of Technology and Engineering, Jingzhou 430023, China

## Abstract

A novel semisupervised extreme learning machine (ELM) with clustering discrimination manifold regularization (CDMR) framework named CDMR-ELM is proposed for semisupervised classification. By using unsupervised fuzzy clustering method, CDMR framework integrates clustering discrimination of both labeled and unlabeled data with twinning constraints regularization. Aiming at further improving the classification accuracy and efficiency, a new multiobjective fruit fly optimization algorithm (MOFOA) is developed to optimize crucial parameters of CDME-ELM. The proposed MOFOA is implemented with two objectives: simultaneously minimizing the number of hidden nodes and mean square error (MSE). The results of experiments on actual datasets show that the proposed semisupervised classifier can obtain better accuracy and efficiency with relatively few hidden nodes compared with other state-of-the-art classifiers.

## 1. Introduction

Recently, ELM [1, 2] shows better performance than traditional gradient-based learning methods and support vector machine (SVM) [3, 4] in regression and classification applications due to its faster learning capacity. As a supervised learning algorithm, the applicability of ELM is seriously restrained [5]. In actual applications, unlabeled data are easy to obtain while the acquisition of labeled data is time consuming and hard. Based on this, it is imperative to extend ELM to achieve semisupervised classification.

Manifold regularization is a frequently used semisupervised learning method based on smoothness assumption [6]. LapRLS [7] and LapSVM [8, 9] based on manifold assumption are frequently used semisupervised learning algorithm. However, manifold regularization is prone to sinking into misclassification in boundary area between several clusters because boundary instances in manifold structure are likely to belong to different classes [10]. Wu et al. [11] proposed semisupervised discrimination regularization (SSDR) for solving misclassification by utilizing discrimination of labeled data in learning. However, due to the scarcity of labeled data, the improvement of misclassification is limited. Wang et al. [12] proposed discrimination-aware manifold regularization (DAMR) in which discrimination of the whole data is considered to improve accuracy. Yet, DAMR merely adopted binary cluster labels which are insufficient for multiclass problem. In view of this, an improved MR framework named clustering discrimination manifold regularization (CDMR) which integrates clustering discrimination of both labeled and unlabeled data with twinning constraints regularization is proposed, and a semisupervised ELM with CDMR framework termed CDMR-ELM is finally developed. The proposed novel framework can effectively avoid boundary misclassification which frequently occurred in manifold regularization and improve the classification accuracy by combining the clustering discrimination with twinning constraints regularization containing lower intracluster compactness and higher intercluster separability.

FOA is a global optimization searching method based on food finding behavior of fruit fly with the advantages of simplicity and being easy to understand [13, 14]. This paper develops an improved variant of FOA named multiobjective fruit fly optimization algorithm (MOFOA) to optimize crucial parameters of CDMR-ELM consisting of the number of hidden nodes and trade-off parameters for further improving the classification accuracy and efficiency. The MOFOA employs MSE to evaluate fitness function and adopts adaptively reduced search area for decision variable to alleviate the possibility of sinking into local extremum and prematurity [15, 16]. Above all, unlike traditional FOA-ELM which implements optimization iteration with fixed number of hidden nodes, MOFOA is based on two objectives: simultaneously minimizing the number of hidden nodes and MSE which can obtain a set of optimal parameters to increase classification accuracy with fewer hidden nodes to reduce computational complexity and enhance efficiency.

The rest of this paper is organized as follows: Section 2 introduces related basic theory.  Section 3 proposes a novel CDMR framework and integrates it with ELM.  Section 4 presents MOFOA to optimize the parameters of CDMR-ELM. Experimental setup and comparison results are given in Section 5.  Section 6 is the conclusion of this paper.

## 2. Related Basic Theory

### 2.1. Extreme Learning Machine (ELM)

Consider a training set containing *N* arbitrary distinct samples {(*x*_*i*_, *y*_*i*_)}_*i*=1,…,*N*_ . Here *x*_*i*_ ∈ *R* ^*d*^ and *y*_*i*_ ∈ *R* ^*m*^, where *d* and *m* represent the dimensions of input and output vector. The output of ELM with respect to sample *x*_*i*_ is determined as follows [17]:

(1) $$\begin{array}{c} f\left(\;x_{i}\;\right)=\overset{L}{\underset{j=1}{\sum }}\beta_{j}G\left(\;a_{j},b_{j},x_{i}\;\right),\;i=1,\ldots ,N, \end{array}$$

where *L* is the number of hidden nodes, *G*(·) is hidden layer output function, and *β*_*j*_ is output weight connecting the *j*th hidden node to output layer. Input weight *a*_*j*_ and bias *b*_*j*_ of hidden nodes are randomly assigned in advance. Equation (1) can be converted into a compact form as follows:

(2) HβY,

(3) H=Ga1,b1,x1⋯GaL,bL,x1⋮⋱⋮Ga1,b1,xN⋯GaL,bL,xNN×L,

(4) β=β1T⋮βLTL×m,

where *H* is output matrix of hidden layer and, by minimizing the square loss of predicted error and norm of weight, ELM analyzes the optimal output weight *β* as follows:

(5) $$\begin{array}{c} \underset{\beta }{\min}\frac{1}{2}{\left\|\;\beta \;\right\|}^{2}+\overset{N}{\underset{i=1}{\sum }}{\left\|\;h\left(\;x_{i}\;\right)\beta -y_{i}\;\right\|}^{2}. \end{array}$$

### 2.2. Manifold Regularization Framework

Manifold regularization framework is built on manifold assumption that close points in the intrinsic geometry of marginal distribution *P*_*x*_ should share similar labels and can effectively solve problem of training dataset consisting of both labeled and unlabeled data [18]. Labeled data {(*x*_*i*_, *y*_*i*_)}_*i*=1,…,*l*_ are generated according to probability distribution *P* and unlabeled data {*x*_*j*_}_*j*=1,…,*u*_ are drawn according to *P*_*x*_ of *P*. By minimizing the following cost function, manifold regularization framework can obtain an optimal classification function *f*(·):

(6) $$\begin{array}{c} \underset{f}{\min}\frac{1}{l}\overset{l}{\underset{i=1}{\sum }}V\left(\;x_{i},y_{i},f\left(\;x_{i}\;\right)\;\right)+\gamma_{A}{\left\|\;f\;\right\|}_{k}^{2}+\gamma_{I}{\left\|\;f\;\right\|}_{I}^{2}, \end{array}$$

where *V*(·) represents loss function and regularization term ‖*f*‖_*k*_^2^ represents the complexity of classifier and regularization term ‖*f*‖_*I*_^2^ which represents smoothness of sample distribution and it can be approximated as

(7) fI212u+l2∑i,j=1l+uwijfxi−fxj2=1u+l2fTLf,

where 1/(*u* + *l*)^2^ is normalization coefficient for the empirical estimate, *L* = *D* − *W* is Laplacian matrix of the whole data, *W* is the weight matrix in which each element *w*_*ij*_ represents the similarity weight between *f*(*x*_*i*_) and *f*(*x*_*j*_), and *D* is a diagonal matrix in which *D*_*ii*_ = ∑_*j*=1_^*l*+*u*^ *w*_*ij*_.

### 2.3. Fruit Fly Optimization Algorithm (FOA)

The steps of FOA are shown as follows.

**Step 1 .**

Randomly initialize the location of fruit fly: *X*_axis, *Y*_axis.

**Step 2 .**

Randomly generate the distance and direction for searching food by using osphresis of an individual: *X*_*i*_ = *X*_axis + RandomValue, *Y*_*i*_ = *Y*_axis + RandomValue.

**Step 3 .**

Estimate the distance *D* between each individual and origin and set the reciprocal of *D* as smell concentration judgment value *S*:

(8) DiXi+Yi,Si=1Di.

**Step 4 .**

Substitute *S*(*i*) into smell concentration judgment function or fitness function of optimization to calculate the smell concentration Smell⁡(*i*) of individual fruit fly: Smell⁡(*i*) = *F*⁡(*S*(*i*)).

**Step 5 .**

Find out the individual fruit fly with maximal smell concentration: $\left[\begin{array}{cc} bestSmell & bestindex \end{array}\right]=\max\left(Smell\right)$ in which bestindex is the location of best individual.

**Step 6 .**

Reserve best smell concentration value and corresponding coordinate of best individual: *F*_best_ = bestSmell, *X*_axis = *X*(bestindex), and *Y*_axis = *Y*(bestindex).

**Step 7 .**

Repeat Step 2 to Step 5 to execute iterative optimization until termination arrived and judge whether the smell concentration is better than previous one, if so, execute Step 5.

## 3. The Proposed Classifier: CDMR-ELM

### 3.1. CDMR Framework

In this paper, we consider a multiclass dataset with *l* labeled data {(*x*_*i*_, *y*_*i*_)}_*i*=1,…,*l*_ and *u* unlabeled data {*x*_*j*_}_*j*=*l*+1,…,*l*+*u*_. Firstly, with the purpose of obtaining the clustering discrimination of the whole data, utilize unsupervised fuzzy clustering method [19] to divide the whole dataset into *C* fuzzy clusters which can effectively reflect the underlying cluster structure. Preserve all cluster labels to form a cluster vector with dimension of (*l* + *u*) expressed as *T* ^*C*^ = [*t*_1_^*c*^,…, *t*_*l*+*u*_^*c*^], where *t*_*j*_^*c*^ which is between 1 and *C* represents the fuzzy clustering label of the *j*th data. In order to fully consider the reliability of clustering result during the learning process, define a membership vector *M* = [*m*_1_^*c*^,…,*m*_*l*+*u*_^*c*^]^*T*^ in which the element *m*_*j*_^*c*^ represents the memberships degradation of the *j*th data defined as follows:

(9) $$\begin{array}{c} m_{j}^{c}=\left\{\begin{array}{cc} 1, & \text{if }x_{j}\text{ is labeled data}\\ \mu , & \text{else}, \end{array}\right.\;\mu \in \left[0,1\right], \end{array}$$

where *μ* is inversely proportional to distance between point and center of corresponding fuzzy cluster. Then, set *K* = *MM* ^*T*^ to describe the reliability of clustering. The clustering discrimination matrix *S* ^*c*^ is defined on the basis of clustering labels and clustering reliability matrix *K*. The element *S*_*ij*_^*c*^ of clustering discrimination matrix *S* ^*c*^ represents whether *i*th instance and *j*th instance belong to the same fuzzy clustering and is defined as follows:

(10) $$\begin{array}{c} S_{ij}^{c}=\left\{\begin{array}{cc} 1, & \text{if }t_{i}^{c}=t_{j}^{c}\\ 1, & \text{if }t_{i}^{c}\neq t_{j}^{c},\;\;K_{ij}\leq 0.5\\ -1, & \text{if }t_{i}^{c}\neq t_{j}^{c},\;K_{ij}>0.5, \end{array}\right. \end{array}$$

where *i*, *j* = 1,…, (*l* + *u*) and *S* ^*c*^ ∈ *R* ^(*l* + *u*)×(*l*+*u*)^. For *l* labeled data, reserve their class labels to form labeled discrimination matrix *S* ^*l*^ as follows:

(11) $$\begin{array}{c} S_{ij}^{l}=\left\{\begin{array}{cc} 1, & if\;y_{i}=y_{j}\\ -1, & if\;y_{i}\neq y_{j}, \end{array}\right. \end{array}$$

where *i*, *j* = 1,…, *l*. Final discrimination matrix *S* ∈ *R* ^(*l* + *u*)×(*l*+*u*)^ is built by combining clustering discrimination matrix and labeled discrimination matrix together:

(12) $$\begin{array}{c} S_{ij}=\left\{\begin{array}{cc} S_{ij}^{l}, & {\text{if }x}_{i},x_{j}\text{ are both labeled data}\\ S_{ij}^{c}, & \text{else}, \end{array}\right. \end{array}$$

where *i*, *j* = 1,…, (*l* + *u*). In summary, *S*_*ij*_ is 1 in two situations: firstly when *i*th instance and *j*th instance belong to the same class for labeled data or the same clustering for unlabeled data and secondly when the reliability of clustering is low.

Further, the optimal solution of classification should possess twinning constraints regularization containing lower intracluster compactness and higher intercluster separability as follows:

(13) minf⁡⁡12∑i,j=1l+ufxi−fxjWc,ij−12∑i,j=1l+ufxi−fxjWs,ij,

where *W*_*c*_ is weight matrix for intracluster in which *W*_*c*,*ij*_ is 1 when *S*_*ij*_ is 1 and *W*_*c*,*ij*_ is 0 when *S*_*ij*_ is −1. *W*_*s*_ is weight matrix for intercluster in which *W*_*s*,*ij*_ is 1 when *S*_*ij*_ is −1 and *W*_*s*,*ij*_ is 0 when *S*_*ij*_ is 1.

Finally, the proposed framework utilizes cluster assumption; that is, data in the same cluster with high similarity weighted by clustering reliability should share the same class label or otherwise possess different class labels. By integrating clustering discrimination of labeled and unlabeled data with twinning constraints regularization described as (13), formulate optimization problem of the proposed CDMR framework as follows:

(14)  minf1l∑i=1lVxi,yi,fxi+γAfk2+γI2l+u2∑i,j=1l+uWijfxi−Sijfxj2+12∑i,j=1l+ufxi−fxjWc,ij−12∑i,j=1l+ufxi−fxjWs,ij

(15)  Wij=Wij0,if  Sij=1KijWij0,else,

where *W* is the weight matrix of the whole data and *W*_*ij*_^0^ represents the similarity between instance *x*_*i*_ and instance *x*_*j*_ according to the distance between them in fuzzy clustering manifold structure and *S* ∈ *R* ^(*l* + *u*)×(*l*+*u*)^ is the final discrimination matrix which integrates fuzzy clustering discrimination with labeled discrimination. In (14), the regularization term (*γ*_*I*_/2(*l* + *u*)^2^)∑_*i*,*j*=1_^*l*+*u*^ *W*_*ij*_(*f*(*x*_*i*_) − *S*_*ij*_ *f*(*x*_*j*_))^2^ represents the fuzzy clustering discrimination of both labeled data and unlabeled data.

### 3.2. CDMR Framework Based ELM (CDMR-ELM)

Based on ELM and the proposed CDMR framework, we construct semisupervised classification model on the basis of CDMR-ELM. Substitute (1) into (14) to obtain the objective function as follows:

(16) argminβ⁡1lHβ−Y2+γAβHHTβT+γI2l+u2βHHTLDHHTβT+γD2βHHTLCHHTβT−1−γD2βHHTLSHHTβT,

where (1/*l*)‖*Hβ* − *Y*‖^2^ is square error of *l* labeled data, *L*_*D*_ = *D* − *W*∘*S* is a Laplacian matrix that is based on clustering discrimination of the whole dataset, *L*_*C*_ = *D*_*C*_ − *W*_*C*_ is a Laplacian matrix for intracluster where *D*_*C*_ is a diagonal matrix denoted by *D*_*C*,*ii*_ = ∑_*i*,*j*=1_^*l*+*u*^ *W*_*C*,*ij*_, and *L*_*S*_ = *D*_*S*_ − *W*_*S*_ is a Laplacian matrix for intercluster where *D*_*S*_ is a diagonal matrix denoted by *D*_*S*,*ii*_ = ∑_*i*,*j*=1_^*l*+*u*^ *W*_*S*,*ij*_.

By zeroing the gradient of the objective function with respect to *β*, convert (16) as follows:

(17) 1lHHβ−YT+γAHHT+γI2l+u2HHTLDHHT+γD2HHTLCHHT−1−γD2HHTLSHHTβT=0.

Then, the solution of the CDMR-ELM is obtained:

(18) β∗=HTHHT+γAI+γI2l+u2LDHHT+γD2LCHHT−1−γD2LSHHT−1T,

where *I* is the identity matrix with dimension of *l* + *u*. According to (1) and (2), the decision function of the proposed semisupervised classification model with regard to input *x* is shown as follows:

(19) fx=hxβ∗=HTHHT+γAI+γI2l+u2LDHHT+γD2LCHHT−1−γD2LSHHT−1T.

Given the hidden weights *a*, biases *b*, and trade-off parameters *γ*_*A*_, *γ*_*I*_, and *γ*_*D*_ previously, the MSE of classification described as follows should be minimized to improve accuracy:

(20) $$\begin{array}{c} \mathrm{MSE}\left(\;a,b,\gamma_{A},\gamma_{I},\gamma_{D}\;\right)=\frac{1}{l+u}\overset{l+u}{\underset{s}{\sum }}{\left(\;y_{s}-f\left(\;x_{s}\;\right)\;\right)}^{2}, \end{array}$$

where *y*_*s*_ represents the predicted output and *f*(*x*_*s*_) represents the actual output for input data *x*_*s*_.

## 4. The Optimized Classifier (MOFOA-CDMR-ELM)

### 4.1. The Multiobjective Optimization Problem and Solutions

Considering that the number of hidden layer nodes strongly influences the semisupervised classification efficiency and training time, the multi-objective optimization problem is to find the optimal SLFNs with a lower MSE and a smaller number of hidden nodes *L* simultaneously as follows:

(21) min⁡ MSEa,b,γA,γI,γD,L,s.t. a,b∈RL×d+1.

The solutions of this multiobjective optimization problem are represented as follows:

(22) a,b,γA,γI,γD,L=a11,…,ad1,a12,…,ad2,…,a1L,…,adL,b1,…,bL,γA,γI,γD,L.

Parameters *γ*_*A*_, *γ*_*I*_, and *γ*_*D*_ can control the reliability of the clustering discrimination from the semisupervised clustering method. If the values of these parameters are larger, the fuzzy clustering discrimination is more important. Otherwise, if the values of these parameters are small, CDMR will degenerate to smoothness assumptions as manifold regularization [12]. Therefore, the values of parameters *γ*_*A*_, *γ*_*I*_, and *γ*_*D*_ should be optimized with the aim of achieving better classification accuracy.

Unlike traditional single-objective optimization problem, optimization problem with multiobjective is impossible to find single solution which simultaneously minimizes all objectives [20–22]. This paper looks for a set of optimal solutions where there is no other efficient solution which improves one element of objectives without deteriorating the remaining elements.

### 4.2. MOFOA-CDMR-ELM Classifier

Considering FOA has possibility of sinking into local extremum and prematurity [15], this paper improves traditional FOA in the following two aspects:

(1) Employing MSE to evaluate fitness function as follows:
  (23) $$\begin{array}{c} {Smell}_{i}=\mathrm{MSE}\left(\;X_{i}\;\right). \end{array}$$
(2) Adopting adaptively reduced search area for decision variable (*a*, *b*) along with iteration going on as follows:
  (24) new_aa±rak,new_b=b±rbk,ra,bk=K−kK2∗rmax,

where *K* is the number of iterations, *r*_*a*,*b*_(*k*) represents the adaptive search area for the *k*th iteration, *k* ∈ [1, *K*] is current iteration index, and *r*_max_ is the maximum search area set as 1/2 which is quarter of gap between high limit and low limit of *a* and *b*.

The algorithm starts with initializing fruit fly swarm *X* ^1^ consisting of* size_of_swarm* individuals represented as vector in (22) in which (*a*, *b*) are randomly assigned from uniform distribution between −1 and 1, (*γ*_*A*_, *γ*_*I*_, *γ*_*D*_) are limited in the range of (2^−24^, 2^24^), and* L* is between 1 and the upper limit for hidden nodes. Next, introduce two variables* add* and* reduce* to control the search of optimal *L* by means of relationship between *L* and MSE. After adjustment of appropriate *L*, evaluate the new solutions and implement* max_it* times inner loop on them for adjusting parameters (*a*, *b*, *γ*_*A*_, *γ*_*I*_, and *γ*_*D*_) in which three trade-off parameters are tuning during the range. Finally, reset the value of* add* and* reduce*. The main loop is repeated *K* times to search global optimal swarm *X* ^*∗*^ in *X* ^*K*^.

The MOFOA for optimizing CDMR-ELM is described as shown in Algorithm 1.

In this paper, we suppose relationship between the MSE and *L* is parabolic or linear. If *L* is proportional to MSE, set* add* to be 0 and set* reduce* to be 1. If *L* is inversely proportional to MSE, set* add* to be 1 and set* reduce* to be 0. If MSE does not improve by increasing or decreasing nodes, set both* add* and* reduce* to be 1. If MSE decreases when *L* both increases and decreases, set both* add* and* reduce* to be 0. Variables* add* and* reduce* guide the search of *L* as shown in Algorithm 2.

In Algorithm 2 *L*_max_, *L*_min_, and *L*_mid_ are maximum, minimum, and middle values of *L* in population *X* ^*k*^, *ω* is uniform random value in (0,1), and *L*′ is the upper limit for hidden nodes.

## 5. Experiment Results and Discussion

### 5.1. Datasets and Experiment Setup

In order to evaluate the accuracy and efficiency of the proposed MOFOA-CDMR-ELM classifier, we perform a set of experiments on several real-world datasets from the UCI machine learning repository and benchmark repository frequently used for semisupervised learning [23]. The details of datasets are shown in Table 1.

Comparison experiments are implemented on two types of classifiers: one type is supervised classifier including SVM and ELM; the other type is semisupervised classifier including SSL-ELM [5, 24], LapRLS [7], LapSVM [8], and the proposed classifier. Divide each dataset into three subsets: testing set, validation set, and training set which is further partitioned into fixed labeled set and unlabeled set. Make sure the labeled set contains at least one sample of each class. Training set is used to train classifiers. Validation set containing labeled data is utilized for optimal model selection. Testing set is used to verify the classifier performance and efficiency. All experiments are implemented in MATLAB 7.0 which is running on a PC with CPU of 3.4 GHZ and RAM of 4.0 GB.

### 5.2. Parameters Setting

For ELM and SSL-ELM, adopt Gaussian function exp⁡(−*b*‖*x* − *a*‖^2^) and use grid search method to find out optimal trade-off parameter *C* between {2^−20^, 2^−19^,…, 2^19^, 2^20^} and number of hidden nodes *L* between {10,20,…, *N*} where *N* is the size of each dataset. For classifiers based on SVM, search optimal parameter *C* between {10^−5^, 10^−4^,…, 10^4^, 10^5^} according to classification accuracy. Furthermore, classifiers based on SVM adopt one-to-rest method to solve multiclass classification problem. The optimal weights *γ*_*A*_ and *γ*_*I*_ of regularization items of LapSVM and LapRLS are searched from grid {10^−5^, 10^−4^,…, 10^5^, 10^6^} by cross validation. The setting of parameters required in the proposed classifier is shown in Table 2.

### 5.3. Effectiveness of the Proposed MOFOA

In order to evaluate the effectiveness of the proposed optimization method in searching for optimal parameters as (23), we compare three classifiers containing the proposed CDMR-ELM, FOA-CDMR-ELM, and MOFOA-CDMR-ELM. Classifier CDMR-ELM with random (*a*_*i*_, *b*_*i*_) can obtain optimal trade-off parameters *γ*_*A*_, *γ*_*I*_, and *γ*_*D*_ by implementing 10-fold cross validation on validation set for 100 times. FOA-CDMR-ELM employs classification error rate to guide the search of optimal weights and biases (*a*_*i*_, *b*_*i*_) as well as weight of regularization items *γ*_*A*_, *γ*_*I*_, and *γ*_*D*_ by giving a fixed number of hidden nodes *L*. MOFOA-CDMR-ELM searches appropriate number of hidden nodes, weights, and biases (*a*_*i*_, *b*_*i*_) as well as weight of regularization items *γ*_*A*_, *γ*_*I*_, and *γ*_*D*_ by simultaneously minimizing MSE and the number of hidden nodes *L*.  Table 3 shows the mean value of classification accuracy and number of hidden nodes by three classifiers on all datasets. Data in bold type represent the optimal classification result and hidden nodes.

From Table 3, we can see that the proposed MOFOA-CDMR-ELM classifier is better than the other two competitive classifiers in 75%. This result fully verifies the effectiveness of the proposed optimization method based on FOA since it adopts adaptively reduced search area for searching in iteration to reduce possibility of sinking into local extremum and premature. Further, focusing on minimizing both MSE and hidden nodes, MOFOA can obtain superior networks with less hidden nodes under the guarantee of better accuracy.

### 5.4. Comparison of Performance

We compare classification accuracy between some state-of-the-art supervised classifiers and semisupervised classifiers on above-mentioned datasets to evaluate efficiency and effectiveness of the proposed classifier.  Table 4 shows the mean value and standard deviation of classification accuracy and Table 5 shows the mean value and standard deviation of running time of all the compared classifiers on 8 datasets.

From Table 4, we can conclude the following:

1. LapRLS and LapSVM outperform supervised classifiers SVM and ELM in semisupervised learning even with a few labeled data, since LapRLS and LapSVM adopt manifold regularization to utilize unlabeled data according to nonlinear geometrical manifold structure embedding in the whole data.
2. Among three existing semisupervised classifiers, by constructing a framework that integrates manifold assumption with constraints between all the labeled data to relieve misclassification in boundary area and enhance the smoothness of decision function, SSL-ELM obtains better classification accuracy than LapRLS and LapSVM.
3. The proposed classifier outperforms SSL-ELM especially on multiclass datasets since it adopts unsupervised fuzzy clustering method and considers inner cluster and intercluster constraints not only between labeled data but also between unlabeled data. Further, the proposed MOFOA plays an important role in enhancing the performance by searching for optimal parameters.

From Table 5, we can see that training time of SVM and ELM is obviously less than semisupervised classifier especially on dataset with large size since they are trained only based on labeled data. To be fair, comparing four semisupervised classifiers trained based on both labeled and unlabeled data, training time of LapSVM classifier for multiclass dataset is more than others. It is possibly due to the fact that one-to-rest method seriously increases running time in iterative process. The proposed classifier optimized by MOFOA obtains optimal parameters in model with high classification and fewer hidden nodes which lead to fast learning speed according to the theory of ELM that the number of hidden nodes is proportional to training time. In general, the proposed classifier can achieve better performance with optimal learning speed.

### 5.5. Performance with Different Number of Labeled and Unlabeled Data

**Figure 1:**
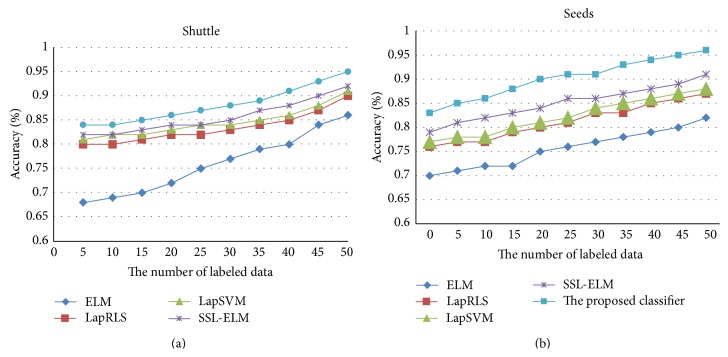
Classification accuracy with respect to different labeled data.

**Figure 2:**
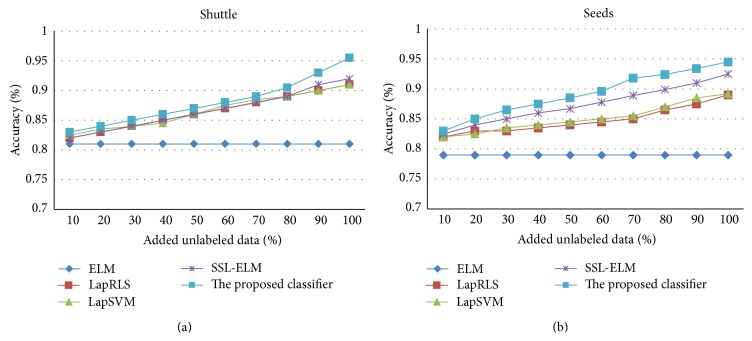
Classification accuracy with respect to different unlabeled data.

The previous experiments are implemented under fixed labeled set and unlabeled set. If the number of labeled and unlabeled data varies gradually, the performance of classifiers exhibits some change tendency.  Figure 1 shows the performance variation of ELM, LapRLS, LapSVM, SSL-ELM, and the proposed classifier on two representative datasets, Shuttle and Seeds, with different number of labeled data by varying proportion of labeled data and unlabeled data in training set.  Figure 2 shows the performance variation of these classifiers with different number of unlabeled data.

From Figure 1, we can observe that, with the increase of number of labeled data, the classification accuracy of every classifier is stably improved. Further, accuracy of the proposed classifier outperforms others all along. From Figure 2, we can see that, with the increase of number of unlabeled data, the classification accuracy of ELM is maintained unchanged since it works only based on labeled data while accuracy of the other semisupervised classifier is enhanced obviously. Further, even with very few unlabeled data, the proposed classifier outperforms SSL-ELM, LapRLS, and LapSVM because it constructs manifold structure by fully utilizing both unlabeled data and labeled data which is effective for supervised learning. In general, the results verify that the proposed classifier can obtain better performance in dynamic semisupervised classification since it integrates discrimination of both labeled and unlabeled data with twinning constraints of fuzzy clusters.

## 6. Conclusion

In this paper, we propose a feasible semisupervised learning method in terms of clustering discrimination of the whole data and twinning constraints regularization named CDMR. Further, we integrate ELM with the proposed semisupervised learning framework to achieve semisupervised classification. With the purpose of enhancing the classification accuracy and training speed of the proposed classifier, we build a novel multiobjective FOA which simultaneously minimizes the number of hidden nodes and MSE to obtain optimal parameters of classifier to guarantee that there are no other SLFNs with higher accuracy and fewer or equal number of hidden nodes. Experiments' results on several datasets confirm the effectiveness and efficiency of the proposed MOFOA-CDMR-ELM classifier. In the future, we will deeply study the sparsity problem of matrix multiplication to further reduce training time.

## Figures and Tables

**Algorithm 1:**
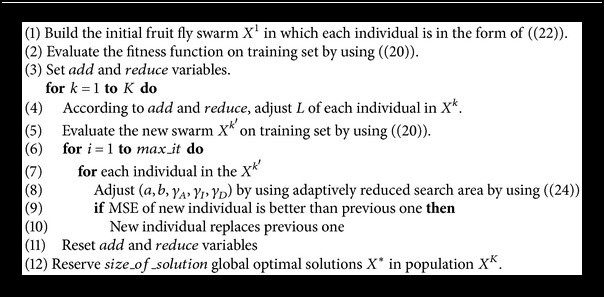


**Algorithm 2:**
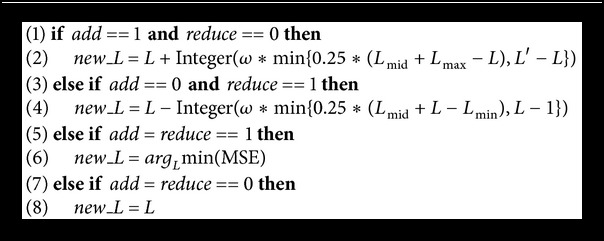


**Table 1:** Details of the selected datasets for semisupervised learning.

Dataset	Classes	Attributes	Size
COIL2	2	1024	1440
COIL20	20	1024	1440
Shuttle	2	9	43500
USPST	10	256	2007
EEG Eye State	2	14	14980
EMGPA	11	8	10000
Seeds	3	7	210
Vertebral Column	2	6	310

**Table 2:** Parameters setting.

Parameter	Value	Description
*size*_*of*_*swarm*	20	Number of individuals in swarm
*K*	20	Number of outer iterations
*max*_*it*	100	Number of inner iterations
*L*′	100	Maximal number of hidden nodes

**Table 3:** Comparisons of average classification accuracy on dataset.

Dataset	CDMR-ELM		FOA-CDMR-ELM		MOFOA-CDMR-ELM	
	Accuracy (%)	Hidden nodes	Accuracy (%)	Hidden nodes	Accuracy (%)	Hidden nodes
COIL2	91.42	34.5	91.86	31.7	**92.41**	**22.1**
COIL20	91.59	39.2	92.23	30.5	**94.57**	**25.0**
Shuttle	92.88	100	93.46	**84.7**	**95.65**	86.9
USPST	91.45	46.9	92.25	79.9	**92.59**	**31.4**
EEG Eye State	**89.58**	100	88.62	**87.6**	89.15	89.2
EMGPA	89.78	97.7	**91.48**	89.5	90.18	**76.5**
Seeds	92.33	29.9	93.21	22.4	**96.88**	**18.3**
Vertebral Column	90.45	27.4	91.65	19.7	**93.76**	**12.8**

**Table 4:** Mean value and standard deviation of classification accuracy.

Dataset	SVM	ELM	LapRLS	LapSVM	SSL-ELM	The proposed classifier
	Accuracy (%)	Accuracy (%)	Accuracy (%)	Accuracy (%)	Accuracy (%)	Accuracy (%)
COIL2	82.11 (±2.33)	83.25 (±2.02)	88.87 (±1.70)	88.52 (±1.45)	90.15 (±1.30)	**92.68 (±2.22)**
COIL20	81.38 (±1.06)	82.88 (±2.34)	87.25 (±2.10)	87.75 (±1.02)	91.38 (±1.50)	**93.15 (±1.67)**
Shuttle	85.28 (±1.85)	85.94 (±1.68)	91.05 (±2.55)	91.25 (±2.04)	92.18 (±2.97)	**95.60 (±2.01)**
USPST	81.45 (±2.95)	81.98 (±1.91)	90.12 (±2.33)	90.38 (±2.19)	91.06 (±2.22)	**93.48 (±1.59)**
EEG Eye State	73.89 (±2.35)	75.13 (±2.58)	85.67 (±1.85)	85.22 (±1.88)	87.50 (±2.44)	**89.55 (±2.10)**
EMGPA	80.44 (±1.87)	81.26 (±2.51)	86.85 (±2.02)	87.30 (±1.52)	88.10 (±1.68)	**91.68 (±2.05)**
Seeds	78.81 (±2.09)	79.63 (±2.65)	86.92 (±2.01)	87.56 (±2.33)	90.25 (±1.32)	**96.65 (±1.72)**
Vertebral Column	82.12 (±1.55)	83.03 (±1.99)	84.68 (±1.96)	84.21 (±1.19)	89.17 (±1.95)	**93.59 (±1.87)**

**Table 5:** Mean value and standard deviation of training time.

Dataset	SVM	ELM	LapRLS	LapSVM	SSL-ELM	The proposed classifier
	Training time (s)	Training time (s)	Training time (s)	Training time (s)	Training time (s)	Training time (s)
COIL2	3.75 (±0.12) × 10^−3^	1.08 (±0.09) × 10^−3^	1.68 (±0.36)	1.55 (±0.08)	0.37 (±0.02)	0.13 (±0.02)
COIL20	3.35 (±0.27) × 10^−2^	1.48 (±0.27) × 10^−3^	2.02 (±0.19)	2.82 (±0.17)	0.51 (±0.08)	0.27 (±0.03)
Shuttle	4.56 (±0.22) × 10^−3^	2.19 (±0.10) × 10^−3^	29.44 (±1.51)	32.38 (±2.90)	12.52 (±3.67)	9.82 (±1.66)
USPST	3.09 (±0.36) × 10^−3^	2.12 (±0.22) × 10^−3^	29.81 (±2.75)	38.35 (±0.20)	5.76 (±0.25)	3.01 (±0.28)
EEG Eye State	3.78 (±0.29) × 10^−2^	2.81 (±0.25) × 10^−2^	26.71 (±3.20)	33.79 (±1.95)	6.36 (±0.96)	5.89 (±0.77)
EMGPA	4.81 (±0.65) × 10^−2^	1.73 (±0.05) × 10^−2^	18.60 (±0.98)	25.23 (±1.65)	6.88 (±0.63)	5.27 (±0.49)
Seeds	2.32 (±0.16) × 10^−4^	5.82 (±0.27) × 10^−5^	7.33 (±0.35) × 10^−2^	8.09 (±0.26) × 10^−2^	3.13 (±0.22) × 10^−2^	2.95 (±0.10) × 10^−2^
Vertebral Column	2.57 (±0.11) × 10^−4^	6.32 (±0.19) × 10^−5^	7.82 (±0.25) × 10^−2^	9.32 (±0.11) × 10^−2^	3.32 (±0.13) × 10^−2^	3.75 (±0.10) × 10^−2^
